# Overreliance on inefficient computer-mediated information retrieval is countermanded by strategy advice that promotes memory-mediated retrieval

**DOI:** 10.1186/s41235-023-00526-6

**Published:** 2023-12-20

**Authors:** Patrick P. Weis, Wilfried Kunde

**Affiliations:** Department of Psychology, Julius-Maximilians-Universität Würzburg, Röntgenring 11, 97070 Würzburg, Germany

**Keywords:** Extended cognition, Technology use, Strategy advice, Strategy selection, Memory formation

## Abstract

With ubiquitous computing, problems can be solved using more strategies than ever, though many strategies feature subpar performance. Here, we explored whether and how simple advice regarding when to use which strategy can improve performance. Specifically, we presented unfamiliar alphanumeric equations (e.g., *A* + 5 = *F*) and asked whether counting up the alphabet from the left letter by the indicated number resulted in the right letter. In an initial choice block, participants could engage in one of three cognitive strategies: (a) internal counting, (b) internal retrieval of previously generated solutions, or (c) computer-mediated external retrieval of solutions. Participants belonged to one of two groups: they were either instructed to first try internal retrieval before using external retrieval, or received no specific use instructions. In a subsequent internal block with identical instructions for both groups, external retrieval was made unavailable. The ‘try internal retrieval first’ instruction in the choice block led to pronounced benefits (*d* = .76) in the internal block. Benefits were due to facilitated creation and retrieval of internal memory traces and possibly also due to improved strategy choice. These results showcase how simple strategy advice can greatly help users navigate cognitive environments. More generally, our results also imply that uninformed use of external tools (i.e., technology) can bear the risk of not developing and using even more superior internal processing strategies.

**Supplementary Information:**

The online version contains supplementary material available at 10.1186/s41235-023-00526-6.

## Introduction

### Using different cognitive strategies

People constantly acquire and process information. Depending on how such acquisition and processing is implemented, performance can substantially vary. In the present study, we refer to different implementations of information acquisition and processing as *cognitive strategies.* For example, to calculate the costs of three croissants at the bakery, one could rely on mental arithmetic. However, the next time, one might have remembered the cost. Instead of mental arithmetic, one can now recall the cost from memory (Compton & Logan, 1991; Logan, 1989). In addition to such *internal* cognitive strategies, one might also use partly environment-based *extended* cognitive strategies for solving arithmetic problems: engaging in smartphone-based calculation, asking other people for help, writing interim solutions on paper, or employing fingers to support counting, just to name a few.

Strategy selection can heavily influence performance. Imagine someone buys three croissants every morning and instead of remembering the price, the person would always engage in a lengthy mental calculation process. Laboratory-based research suggests that some people indeed prefer calculation over recall even after numerous encounters of the task solution (Bourne et al., 2010). Why would the person not recall frequently observed information from memory? We argue that there are two options. Either the person (1) never established a functional memory trace of the corresponding solution in the first place or (2) established the trace but decided for the well-known calculation strategy instead of direct solution retrieval. With the present research, we investigated to which extent strategy advice is feasible to encourage participants to employ more efficient retrieval strategies and drop inefficient calculation strategies and to which extent such advice facilitates the formation or retrieval of task solutions.

### Establishing and using an internal retrieval strategy

Without having a task’s solution encoded in memory, there is obviously no way to rely on memory retrieval to solve the task. Contrarily, once an internal memory trace has been established and remains accessible, memory recall is an incredibly efficient way to solve a wide variety of tasks. As indicated by the bakery example, retrieving “5.40 EUR” from memory is fast and would render the calculation of “3 × 1.80 EUR” unnecessary. But how can human performers create such an accessible memory trace? First, a sufficient number of re-encounters of a specific problem at hand is mandatory. Only if a specific problem had occurred before, can the solution to that problem be encoded in memory. The required number of repetitions enabling retrieval can be pretty low though, sometimes being limited to just one previous encounter (Frings et al., 2020). Moreover, it has been suggested that the number of retrieval attempts fosters subsequent retrieval (Karpicke & Roediger, 2008; Logan & Klapp, 1991).

Unfortunately, human performers often engage in problem solving without even trying to retrieve previous solutions, which undermines the development of a retrieval-based strategy (Pyke & LeFevre, 2011). Accordingly, advising participants to engage in retrieval attempts in a 2-s-window designated for that purpose before using another strategy to solve the problem facilitated direct retrieval in the future (Pyke & LeFevre, 2011). That retrieval attempts are optional is also suggested by another study: even after establishing direct retrieval for some solutions in a learning phase, a third of participants nearly exclusively (> 95%) relied on externally retrieving the solution on a computer screen via mouse movements in a test phase (Weis & Wiese, 2019). Thus, even though these participants had accessible memory traces of many solutions as indicated by reaction times during the test phase, they decided to still look up the solution on the computer before providing their response. Because participants who heavily (> 95%) relied on external retrieval during the test phase exhibited no influence of a problem’s learning frequency during the learning phase on mouse movement onset during the test phase, it is highly likely that these participants indeed did not engage in retrieval attempts before starting to use the mouse (Fig. 7 in Weis & Wiese, 2019), which excludes the possibility that participants used computer-based strategy only to double-check the results in a sequential manner (Hecht, 2006; Siegler, 1988). Similarly, another study suggests that even with substantial practice, participants are in voluntary control of whether or not to use a cognitive strategy, including internal retrieval (Haider et al., 2005).

Thus, first, it seems likely that participants do not automatically engage in retrieval attempts when encountering a problem, which might impede the creation of memory traces and thus the future use of internal retrieval. Second, even if fast internal retrieval had already been established, participants seem to be able to decide to not use this—very efficient—internal retrieval strategy. A corollary is that encouraging retrieval attempts can be beneficial in two ways: (1) to create and improve an internal retrieval strategy in the first place and (2) to actually use that strategy after creation.

### Giving strategy advice

As already implied above, giving specific advice regarding which cognitive strategies to use when seems to be a promising route to improve performance. This has been suggested for advice based on rules presented before the first trial (e.g., always try internal retrieval first; Pyke & LeFevre, 2011) or as specific advice which strategy works better right before each trial (Gilbert et al., 2020). Similarly, advice can also help problem solvers perform novel cognitive strategies. For example, while young children with high working memory are more likely to spontaneously use a finger counting strategy, children with low working memory benefit from being taught this specific strategy (Dupont-Boime & Thevenot, 2018). Analogously, instructions to act out or talk through a written dialogue help performers memorize the dialogue when compared to a condition that only urges performers to do whatever helps them memorize the dialogue (Noice & Noice, 2001). Maybe most famously, instructions that help us piggyback on our spatial abilities to memorize facts can drastically improve memory performance (Dresler et al., 2017; Maguire et al., 2003).

In sum, strategy advice has a good track record of improving performance in cognitive tasks. One might say that the benefit of strategy advice is obvious. We would respond that human performers are known to exhibit quite adaptive strategy choices already without strategy advice (for reviews, see, e.g., Anderson, 1990; Gray et al., 2006; Gilbert et al., 2022), which sets the bar for improvement at substantial height. Accordingly, another seemingly obviously beneficial intervention not always surpasses this bar: giving performance feedback has the potential to corrupt adaptive strategy choice rather than support it (Engeler & Gilbert, 2020; Weis & Kunde, 2023b). Specifically, participants in both studies received performance feedback after solving problems with different cognitive strategies. Feedback substantially improved how accurately performance was estimated with both strategies. However, in subsequent choice trials, participants in a control group without feedback in the first place were making descriptively more rather than less adaptive choices between the strategies. Thus, seemingly beneficial explicit interventions seem to have the potential to meddle with rather than support strategy choices that were based on implicit processing.

### Current study

**Fig. 1:**
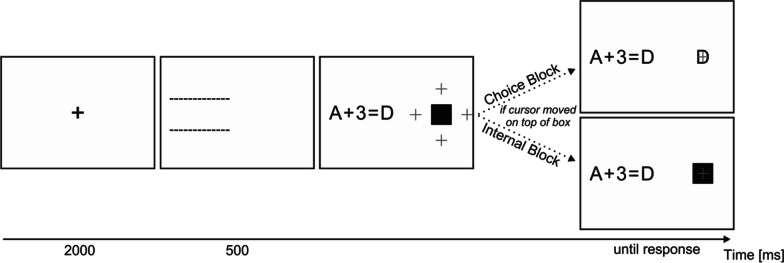
Extended Alphanumeric Paradigm. *Note*. The paradigm is an extension of the classic alphanumeric paradigm (Compton & Logan, 1991; Logan & Klapp, 1991; also see Zbrodoff, 1995) and was already used in earlier research (Weis & Wiese, 2019). Participants have to solve alphanumerical problems of the format “letter + number = letter” and asked to indicate whether counting the indicated number up the alphabet from the former letter equals in the latter letter. For example, counting up the alphabet from A to D would inform the participant that “*A* + 3 = *D*” is a correct while “*A* + 3 = *E*” is an incorrect equation. Crucially, participants can over time transition from a slow internal counting to a fast internal retrieval strategy. In contrast to the classic paradigm, participants can in addition to solving alphanumeric equations by (1) counting up the alphabet [counting strategy] or (2) retrieving the correct solution from memory [internal retrieval strategy], also (3) move a mouse cursor—which spawns at one of four locations next to a black box—on top of that box that would then reveal the correct solution [external retrieval strategy]. Participants receive a 500 ms feedback when committing errors; the 2000 ms ITI would then be shortened accordingly

**Fig. 2:**
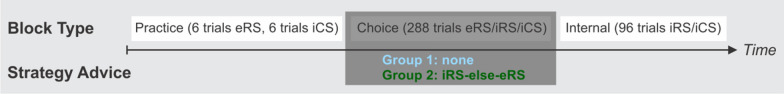
Procedure. *Note*. The practice block could be voluntarily repeated and had to be repeated if the mouse had not been used during external retrieval trials. *eRS* external retrieval strategy, *iCS* internal counting strategy, *iRS* internal retrieval strategy

So far, we have been arguing that strategy advice should be a promising way to (1) help performers establish cognitive strategies as well as (2) help performers use these strategies in the right occasions. Here, we tested these conjectures with participants naive to the problems at hand and with both internal and environment-based cognitive strategies available, which we think mimics real settings in a highly technologized world to a considerable degree. Specifically, we asked participants to validate alphanumeric equations either without or with strategy advice while measuring their performance. Alphanumeric problems like “*A* + 3 = *D*” can be validated by checking whether counting the alphabet up for three letters, starting with *A*, equals *D* (Fig. 1). Crucially, participants were able to use three cognitive strategies associated with different performances to validate the equations. Two strategies were immediately available and one needed to be established with practice. Initially, participants were able to count up the alphabet as indicated before (internal counting strategy, iCS) or use the mouse and hover the cursor over a black box that would then reveal the correct answer which could then be used to validate the equation (external retrieval strategy, eRS). Since equations were repeated frequently, participants could over time learn the validity of the equations by heart (internal retrieval strategy, iRS). Once internal memory had been sufficiently established, findings from a previous study suggest that counting is the slowest, followed by external retrieval, and then internal retrieval is the quickest (Weis & Wiese, 2019). After being able to freely choose between strategies in a choice block, we tested our participant’s performance with exclusively internal strategies in an internal block (Fig. 2).

**Fig. 3:**
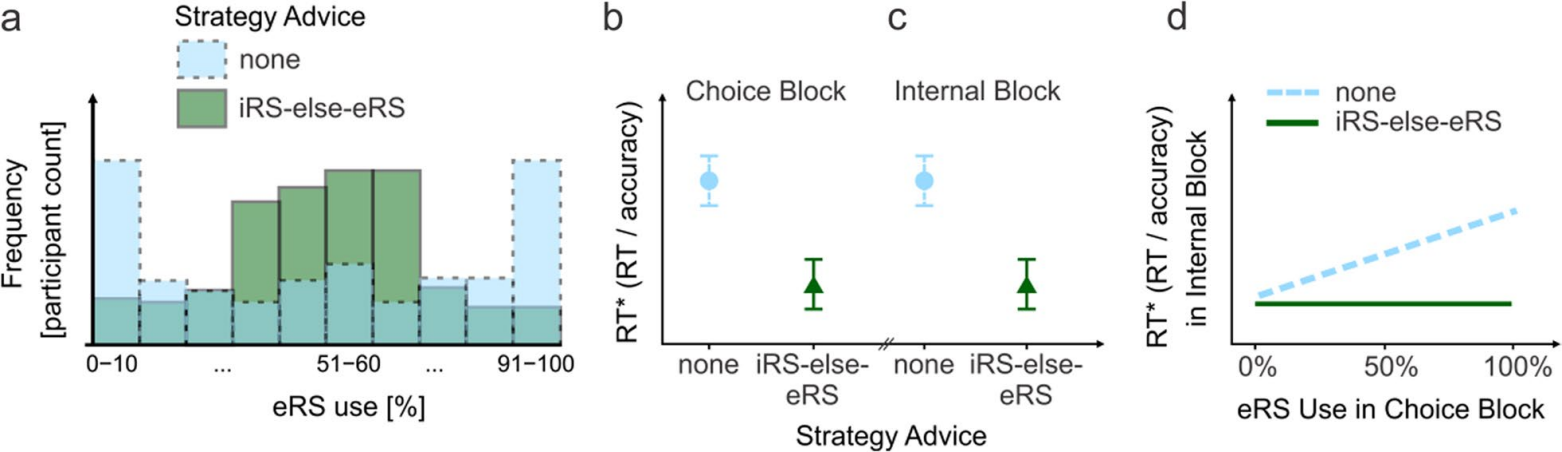
Expected Results. *Note*. Expected results in **a** depict a situation where external retrieval strategy use during the choice block is descriptively different in the no advice in comparison with the advice group. Specifically, the extreme use of only internal or only external strategies that was observed in an earlier study (Weis & Wiese, 2019) should be less pronounced with advice. Expected results in **b** indicate that the altered strategy use during the choice block as depicted in **a** is associated with improved performance. Expected results in **c** indicate that the beneficial effects of strategy advice carry over to the internal block and are associated with establishment of a more efficient internal retrieval strategy (**d**). Note that the results expected in **a**–**c** were preregistered, whereas **d** was added after preregistration because our initially planned analysis proved to be inapplicable for the question at hand; see https://osf.io/r8fb3

Specifically, we investigated whether advising an *iRS-else-eRS advice* group to first try internal retrieval (i.e., the iRS) before engaging in external retrieval (i.e., the eRS) will improve performance also through improved memory or only through improved strategy choice, or not at all, compared to a *no advice* control group without specific instructions regarding strategy choice. As a first step, we hypothesized that advice does indeed improve performance as compared to no advice in the choice block (Hypothesis H1) because the less efficient counting strategy will be entirely skipped in favor of the better performing (Weis & Wiese, 2019; also see Compton & Logan, 1991; Pyke & LeFevre, 2011) internal and external retrieval strategies. With two choice options, participants frequently exhibit a bimodal distribution of strategy use frequency. That is, most participants either almost always, or almost never, use a specific strategy (Weis & Kunde, 2023a; Weis & Wiese, 2019). Confirmation of H1 would be a first indicator that strategy advice is suited to descriptively break this perseveration strategy (Fig. 3a) and to improve overall performance (Fig. 3b). As a second step, we hypothesized that the potential benefit caused by strategy advice in the choice block carries over to an internal block in which all participants received identical instructions. In other words, with *iRS-else-eRS* advice in the choice block, performance in the final internal block should remain overall superior as compared to the no advice group (H2; Fig. 3c). Confirmation of H2 would indicate that strategy advice can have long-lasting effects and outlast situations in which explicit advice had been given. But why exactly would the effect outlast situations in which the advice is present? We hypothesized that this was at least partially caused by increased efficiency of internal retrieval rather than by improved strategy choice alone (H3). In other words, we speculated that the effect of advice—which was presented in the choice block only—carried over to the internal block because the advice helped participants establish better internal memory traces—i.e., improve internal retrieval. Using reverse logic, this confirmation of H3 would also mean that establishment of internal memory traces does not happen automatically without advice. We tested this conjecture by analyzing whether participants in the other condition—i.e., the no advice condition—with an increased external retrieval use during the choice block exhibited decreased performance in the internal block because they missed out on opportunities to build up internal memory (Fig. 3d).

## Methods

### Participants

Data collection stopped once 102 participants or 51 participants per group could be included following preregistered exclusion criteria.^1^ Sample size was based on a power estimation conducted in the software G*Power based on a medium effect when using a one-sided independent *t*-test (*d* = 0.5, alpha = 0.05, 1–beta = 0.80; Faul et al., 2007). The effect size *d* was adopted as a conservative guess based on a more powerful but similar advice manipulation (*η*_p_^2^ = 0.51, which approximately equals *d* = 0.5 after conversion, for reaction time differences in Pyke & LeFevre, 2011). Two participants were excluded because they were outside of 2.5 standard deviations around the condition’s RT mean (both no advice group), and 24 participants were excluded because they scored below 75% accuracy in the internal block (eleven no advice group, thirteen advice group). In addition to the preregistered criteria, we decided to exclude four participants (one no advice group, three advice group) because they were outside of 2.5 standard deviations around their group’s RT* mean in either the choice or the internal block, leading to a final sample size of 98 participants (50 no advice group, 48 advice group; mean age 26.7 years; age range 18–54; 74 female, 24 male). We decided for this additional procedure to remove highly biasing observations while still sticking close to the preregistered plan.

### Apparatus

The experiment ran on a BENQ XL2411P 24-inch monitor set to a resolution of 1920 × 1080 pixels and a refresh rate of 100 Hz. The screen was positioned about 75 cm in front of participants. The experiment was programmed in and run with MATLAB version R2016a (The Mathworks, Inc., Natick, MA, USA) and the Psychophysics Toolbox (Brainard, 1997; Pelli, 1997). Participants responded using a USB-connected standard keyboard and mouse.

### Procedure and task

After being welcomed in our laboratory, participants provided informed consent and received screen-based instructions regarding the task and all three possible cognitive strategies that can be used to solve the task. Each strategy was introduced with a text-based walk-through. Participants then engaged in twelve practice trials which they were asked to solve with either the counting or the mouse strategy immediately before each trial. Problems used for practice trials were not re-used for the main experiment. Practice trials followed the same structure as the choice trials of the main experiment. Feedback was presented only if an incorrect answer was given. If participant did not use the mouse in at least four trials or they indicated that they wanted to redo the practice, the twelve practice trials started over again. When participants indicated that they understood the task and do not need more practice, the advice manipulation occurred via both screen-based and paper-based instructions. Specifically, participants in the iRS-else-eRS advice group were asked in German to “Please always try to retrieve the solution from memory (alternative 2). If that does not work, please use the mouse instead (alternative 3). Please avoid counting (alternative 1). Please try to answer as quickly and correctly as possible […]”. Participants in the no advice group were asked in German to “Please try to use the three alternatives, counting (alternative 1), memory (alternative 2), and mouse (alternative 3) in a way that enables you to answer as quickly and correctly as possible.”. All participants were told that “We are able to use your data only if you try following these instructions”. Participants were explicitly asked whether they understood the instructions and only allowed to continue if they agreed. Participants then engaged in choice trials with a self-paced break every 30 trials in which they were reminded of the instructions according to their advice group. After completing 288 choice trials, all participants were asked in German to “Please do not use the mouse for the remaining problems (the black box is deactivated). Please use your mental abilities (memory or counting) instead.” Participants then engaged in 96 internal trials. The procedure is illustrated in Fig. 2. Please note that the advice manipulation took place exclusively in the choice block. The session concluded with demographic questions, several questions about strategy preference and reasons for strategy choice, and the German Need For Cognition Scale (Bless et al., 1994; Cacioppo & Petty, 1982); see Additional file 1 for the full list of questions. On average, the whole experimental procedure took 45 min and was paid at an hourly rate of 10 Euros.

### Stimuli

After practicing with unique equations in the practice block, each participant encountered each of the 24 following equations four times in each sub-block consisting of 96 trials in fully randomized order: "*B* + 4 = *F*", "*M* + 5 = *S*", "*H* + 4 = *L*”, “*M* + 5 = *R*”, “*O* + 3 = *S*”, “*A* + 3 = *D*”, “*P* + 5 = *U*”, “*C* + 5 = *I*”, “*C* + 5 = *H*”, “*K* + 3 = *N*”, “*F* + 5 = *K*”, “*P* + 5 = *V*”, “*H* + 4 = *M*”, “*A* + 3 = *E*”, “*L* + 4 = *Q*”, “*N* + 4 = *S*”, “*K* + 3 = *O*”, “*B* + 4 = *G*" "*L* + 4 = *P*”, “*O* + 3 = *R*”, “*D* + 3 = *G*”, “*N* + 4 = *R*”, “*D* + 3 = *H*”, “*F* + 5 = *L*". The choice block consisted of three consecutive sub-blocks and the internal block of one sub-block. Each left-hand side of an equation occurred eight times in each sub-block: four times with the correct right-hand side and four times with an incorrect right-hand side, which always was the one letter further up the alphabet than the correct right-hand side.

### Analyses

#### Data cleaning

All practice trials as well as all trials further than 2.5 SD from individual RT means of the respective block (2.6% of all non-practice trials) were excluded from all analyses, including accuracy-based analyses. For RT-based analyses, only correct trials were used. RT* was calculated by dividing each correct RT by the mean accuracy of that participant with problems of the same addend size (i.e., 3, 4, or 5) in the respective block (RT* is also known as the Inverse Efficiency Score; Townsend & Ashby, 1978). For example, correctly responding to a problem with addend size 3 after 1000 ms in a specific trial and an accuracy of 90% in all trials with addend size 3 of that participant would result in a RT* of 1111 ms in that trial.

#### Analyses

For improved readability, all analyses are explained in more detail at the respective location of the Results section. Broadly, H1 and H2 were analyzed with independent *t*-tests and H3 was analyzed with hierarchical linear regression. Whenever we report confidence intervals of effect sizes, we refer to bias-corrected and accelerated intervals that were computed with R’s bootES package (version 1.2.1).

## Results

### eRS use proportion

**Fig. 4:**
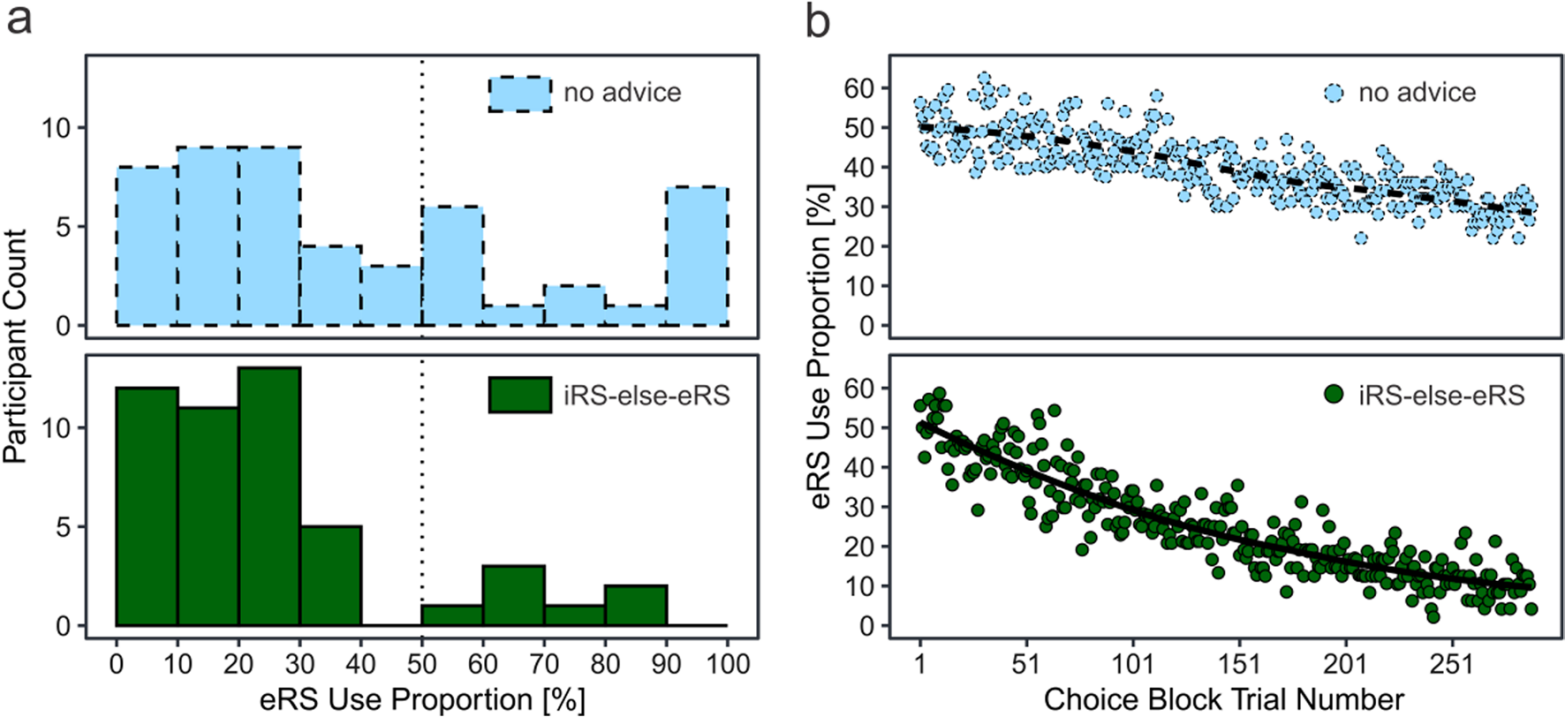
External retrieval during choice block. *Note*. Some participants without advice nearly exclusively relied on external retrieval (**a**). A trial was defined as solved with the external retrieval strategy if the mouse had been hovered on top of the box and the correct solution had been revealed. In both advice groups, participants relied less on external retrieval over time (**b**). The decline was more pronounced in the iRS-else-eRS advice group than in the no advice group (Statistically, the decline was evident in two exploratory independent *t*-tests. Participants of the advice and the no advice group used the eRS similarly frequently throughout the first five trials *M*_advice_ = 50.1%_,_ *M*_no advice_ = 50.6%*, t*(96) = .06, *p* = .954*, d* = .01, CI95_*d*_ = [− .39 .39], but not during the last five trials, *M*_advice_ = 10.4%_,_ *M*_no advice_ = 30.4%,* t*(96) = 3.11, *p* = .002*, d* = .63, CI95_*d*_ = [.23 .98], of the choice block.). Each dot represents the average proportion of eRS at any given trial in the choice block. eRS = external retrieval strategy, iRS = internal retrieval strategy

Without advice, participants in a previous study with the same paradigm frequently either preferred internal strategies or external retrieval, but the minority of participants used both (Fig. 1a; Weis & Wiese, 2019; see Weis & Kunde, 2023a, for similar findings with other paradigms), resembling a bimodal distribution. Thus, as a first indicator that advice affected strategy choice, we expected a bimodal distribution for the no advice group in comparison with a less bimodal distribution in the iRS-else-eRS advice group (Fig. 3a). Results confirm this expectation in so far as the advice group relied less on external retrieval (Fig. 4a). However, to our surprise, a considerable number of participants in the advice group rarely used external retrieval throughout choice trials. This seems to be incompatible with their instruction (attempt internal retrieval first, then external), because internal retrieval is possible only after a minimum number of encounters of correct solutions. Therefore, we took a closer look at the first twelve trials in which participants should—despite the randomized presentation—be mostly unable to retrieve any correct solution from memory. Results indicate that many participants did initially not sufficiently rely on external retrieval to obtain their answers (Additional file 1: Fig. S1). Here, we used a liberal criterion of 33.3% or 4 out of 12 trials in which participants needed to employ external retrieval to be categorized as *following advice instructions*. Based on this criterion, a substantial number of participants, sixteen participants or 33.3%, did not follow instructions. Since these participants still scored an accuracy of 87%, they must have relied on the internal counting strategy rather than chance. Thus, for the remainder of the analyses, it should be kept in mind that despite a most thorough implementation^2^ of the advice instruction, some participants initially did not follow the advice. However, these sixteen participants that initially hardly relied on external retrieval likely still at least partially obeyed the iRS-else-eRS advice later in the study; for details consult the caption of Additional file 1: Fig. S2.

Even though our advice might not have succeeded in suppressing internal counting for all participants, differences in strategy use started to emerge over time when looking at the sample as a whole. These differences can be best seen when looking at how frequently participants hovered over the black box, which indicated external retrieval. While both groups relied less on external retrieval over time, this decline was more pronounced in the advice group (Fig. 4b), which ultimately led to the different distribution depicted in Fig. 4a.

#### Performance

**Fig. 5:**
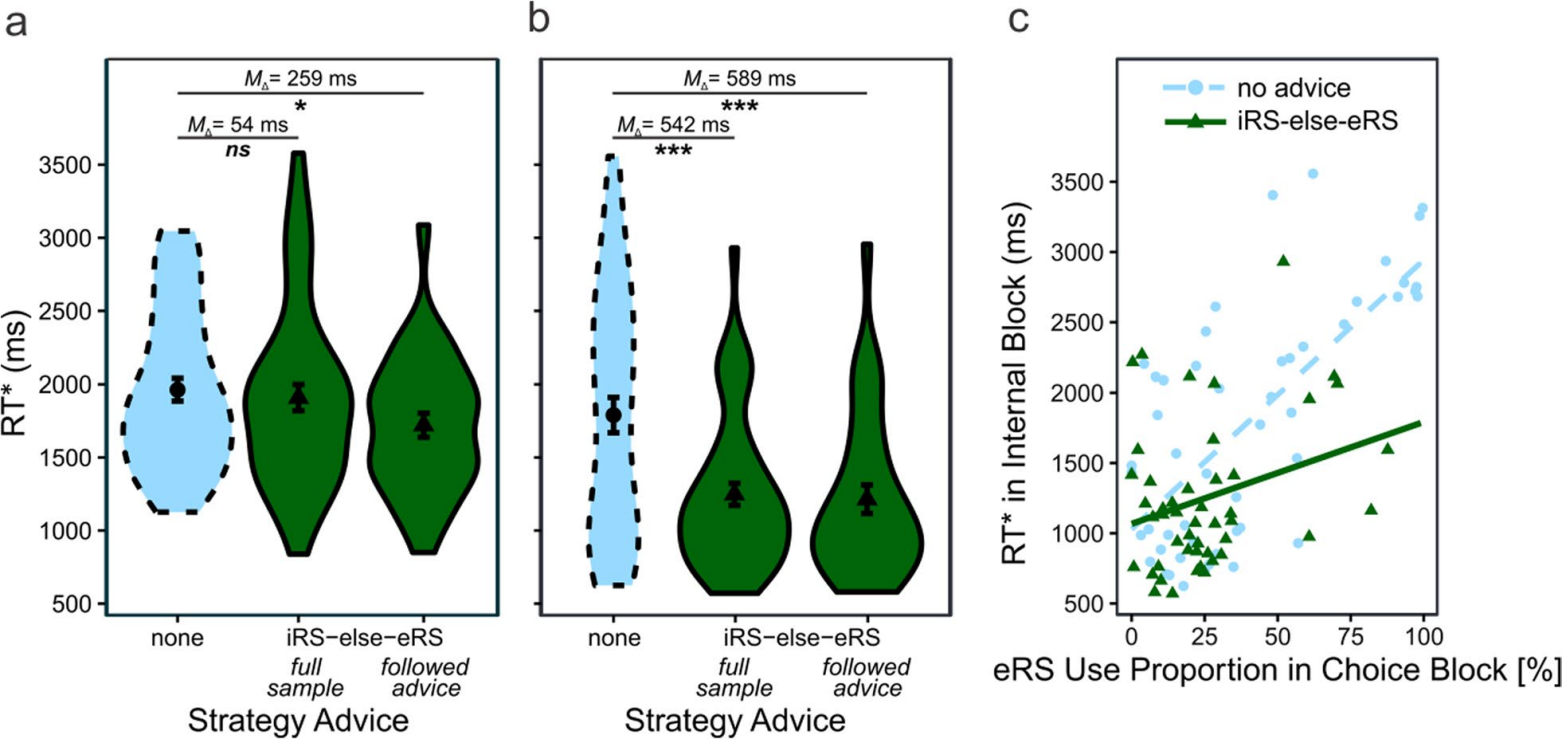
Performance in choice and internal test blocks. *Note*. Participants in the iRS-else-eRS advice group outperformed the no [‘none’] advice group both in the choice block (**a**) and the internal block (**b**). Linear regressions suggest that iRS-else-eRS advice in comparison with no advice improved internal memory encoding during eRS trials (c; see significant interaction term in Table 1)

Descriptively, iRS-else-eRS advice led to altered strategy use over time in the choice block (Fig. 4b). Is this change associated with improved performance? A one-sided independent *t*-test for RT* differences between advice groups in the choice block suggests otherwise; *M*_advice_ = 1909 ms_,_ *M*_no advice_ = 1963 ms, *t*(96) = 0.45, *p* = 0.325, *d* = 0.09, CI95_*d*_ = [− 0.30 0.49] (Fig. 5a). However, RT* differences emerged when excluding the sixteen participants who disobeyed advice instructions; *M*_advice_ = 1704 ms_,_ *M*_no advice_ = 1963 ms, *t*(80) = 2.23, *p* = 0.014*, d* = 0.50, CI95_*d*_ = [− 0.06 0.93].^3^ We conclude that iRS-else-eRS advice can indeed improve performance during the choice block, which confirms H1. However, we also conclude that some participants have strong preferences and might not or only partially implement strategy advice.

Did advice alter performance in the subsequent internal test block when external retrieval was no more available? A one-sided independent *t*-test for RT* differences between advice groups provided strong evidence for this claim; *M*_advice_ = 1248 ms_,_ *M*_no advice_ = 1790 ms, *t*(96) = 3.77, *p* = 0.0001, *d* = 0.76, CI95_*d*_ = [0.36 1.15] (Fig. 5b).^4^ Thus, strategy advice—if obeyed—not only proved beneficial when the eRS was available but continued being beneficial once the advice manipulation ceased and the eRS started being unavailable, which confirms H2.

#### Reasons for performance benefit caused by strategy advice

Did participants in the advice group simply choose to rely more on internal retrieval than counting in the internal block? Or did advice additionally help them to improve internal retrieval? We specifically looked at eRS trials in the choice block to investigate these questions. Without advice, eRS trials should contribute less to memory traces and thus iRS performance than with advice. With advice, however, future iRS performance should be strengthened in the same trial in which an eRS is employed. Consequently, we investigated whether the amount of eRS use in the choice block negatively impacted performance in the internal block selectively for the no advice group. To this cause, we employed a hierarchical linear regression. First, as a baseline, we predicted internal block performance (RT*) based on advice group and eRS use proportion in the choice block. We then added the interaction between advice group and eRS use proportion in the choice block in a second step and tested the incremental R^2^.

Results indicate that adding the interaction term improved prediction of internal block RT*; *F*(1) = 6.5, *p* = 0.012, Δ*R*^2^_adj_ = 3.2% (Table 1). The interaction is depicted in Fig. 5c: Only participants without strategy advice exhibited poor internal retrieval performance when they frequently used external retrieval before; participants with strategy advice exhibited comparably good internal retrieval performance even if they frequently used external retrieval before. Specifically, a 100% increase in *eRS use proportion in choice block* was associated with a 1189 ms lower increase in RT* for the advice in comparison with the no advice group. In sum, these results suggest that the performance benefit caused by strategy advice was at least partially due to increased efficiency of the iRS rather than only due improved strategy choice, which confirms H3.

**Table 1:** Hierarchical linear regression predicting RT* during internal block

Variable	Step 1 (baseline)			Step 2 (with interaction)		
	*B*	SE	*p*	*B*	SE	*p*
Constant	1177 ms	121 ms	< .0001	1029 ms	131 ms	< .0001
eRS use proportion during choice block	1543 ms	222 ms	< .0001	1915 ms	261 ms	< .0001
Strategy advice	− 313 ms	122 ms	.012	− 38 ms	182 ms	.833
eRS use proportion during choice block × strategy advice				− 1189 ms	466 ms	.012
*R*^2^_adj_	.410			.442		
Δ*R*^2^_adj_				.032 *		

We did not center the continuous predictor because a value of “0” eRS use proportion during choice block can be meaningfully interpreted. We dummy-coded the categorical predictor strategy advice, with no advice coded as 0 and iRS-else-eRS advice coded as 1. Step 1 has 95, Step 2 94 degrees of freedom

*p = .012

We conducted an additional exploratory analysis to further test H3, i.e., whether iRS-else-eRS advice improved internal memory rather than merely improved strategy choice. Previous research suggests that with only internal strategies available, a cutoff value of 2000 ms can reasonably well be used to distinguish whether a participant employed iRS or iCS (Compton & Logan, 1991). Following that rationale, trials with RT*s below 2000 ms were likely answered with iRS and trials with RTs above 2000 ms were likely answered with iCS. Now, if iRS-else-eRS advice improved internal memory, participants in the iRS-else-eRS advice group should be faster than the no advice group when only looking at trials with an RT below 2000 ms. No difference should emerge for trials with an RT* above 2000 ms because counting proficiency should not be affected by the present strategy advice. Using independent *t*-tests, this is what we found. In internal block trials below 2000 ms, participants in the advice group were faster than in the no advice group, *M*_advice_ = 1045 ms_,_ *M*_no advice_ = 1191 ms, *t*(94) = 2.47, *p* = 0.015*, d* = 0.51, CI95_*d*_ = [0.12 0.93]. No such effect was found for internal block trials above 2000 ms, *M*_advice_ = 3362 ms_,_ *M*_no advice_ = 3377 ms, *t*(43) = 0.08, *p* = 0.935*, d* = 0.03, CI95_*d*_ = [− 0.82 0.65]. Note that for this analysis we excluded all participants who did not correctly complete at least two trials for each of the three addends (2 participants for the < 2000 ms analysis, 53 participants for the > 2000 ms analysis^5^). In sum, this exploratory result shows that advice specifically improves performance of internal trials with an RT* below 2000 ms, which excludes trials in which the iCS had been employed. Thus, the result further supports an improved establishment of memory traces as the mechanism underlying the advice-induced performance benefit.

Note that we also report our preregistered analysis to investigate reasons for performance benefits caused by strategy advice in the SM (Additional file 1: Fig. S3). Despite confirming the corresponding hypothesis, we dropped this analysis due to lack of interpretability. For further information, please consult the SM.

## Discussion

In the present study, participants were situated in a context in which they needed to solve well-confined problems with one of three cognitive strategies: internal retrieval, internal counting, or external retrieval. Our results indicate that advising our participants when to best use which strategy substantially improved performance. Conversely, participants without strategy advice employed strategies in a suboptimal manner despite the rather simplistic context of this laboratory study when compared to real-life situations. Specifically, without advice, participants had trouble establishing or retrieving internal memory relevant for task solution. This trouble was at least partially caused by the use of the computer-mediated external retrieval strategy which seemed to tamper with memory formation. Crucially, advising participants to try internal memory retrieval right before each use of the computer-mediated external retrieval enabled memory formation again. Interestingly, the retrieval attempts seemed to have had little costs attached to them since strategy advice led to improved performance already in the choice block. In contrast, an earlier study that found long-term benefits of retrieval attempts reserved a 2 s window in each trial for retrieval attempts (Pyke & LeFevre, 2011). Based on the present results, we argue that such a window is not even necessary and that performers can implement internal retrieval attempts *on-the-fly*: Simple strategy advice can be enough to create and employ strategies in a more adaptive manner.

However, despite the large potential benefits of strategy advice, we want to emphasize that some participants might be reluctant to follow such advice. Here, about a third of the advice group only partially followed advice and paid with poorer short-term performance in the choice block. Even though it seems likely that these participants followed advice later on during the choice block, ultimately leading to good performance in the internal block, we conceive compliance to be an important issue that deserves further research.

### Causes for suboptimal performance without advice

Suboptimal performance of the no advice group might be rooted in suboptimal strategy choices. Specifically, some participants might choose not to rely on internal retrieval in a certain trial. That participants have a choice whether or not to use internal retrieval is somewhat surprising from the perspective of episodic retrieval accounts of human performance. These accounts hold that individual problems are stored, even after a single encounter, with the correct response to that problem, with later encounters of the same problem prompting a more or less automatic retrieval of the previous response (Logan, 1989). If such retrieval was actually performed, the performance of the no advice group suggests that there is at least considerable control about whether or not to use the retrieved response. Thus, participants in the advice condition (try internal retrieval first) might have more willingly embraced the existing wealth of internally stored information. Alternatively, our participants might have had enough metacognitive control to completely skip retrieval in the first place (Bajic & Rickard, 2009; Bourne et al., 2010; Haider et al., 2005).

Suboptimal performance of the no advice group might also be rooted in a less efficient buildup of internal memory. One might argue that using computer-mediated strategies to find solutions does not preclude establishment of internal memory. After all, task and solution are visible during external retrieval, which could lead to automatic encoding of each instance or episode (Frings et al., 2020; Logan, 1989). However, present and earlier (Pyke & LeFevre, 2011) results suggest that retrieval attempts before the use of external retrieval are more effective in that respect. In other words, we suggest that in the present paradigm, solution- or response-oriented encoding of instances/episodes is not automatic but benefits from volitionally enforced internal retrieval attempts. Relatedly, previous research suggests that participants strategically decrease encoding of items that can be put in an external store, if and only if that store was perceived as reliable (Storm & Stone, 2015). In sum, it seems that establishing and improving internal retrieval is no automatic byproduct of using tech-mediated external retrieval. Instead, users of external retrieval strategies can benefit from making well-informed decisions about whether or not to establish internal memory in parallel to using an external strategy.

### Is external retrieval bad for us?

If external retrieval can lure us away from more efficient strategies and even tamper with memory formation, this sounds like bad news. This pessimistic view is supported by a decrease of arithmetic fluency after calculators had been introduced into the educational system (LeFevre et al., 2014). But we by no means argue that external retrieval of information is bad or should be avoided in general. Instead, we argue that not all performers use external retrieval in the most beneficial way. We have shown that, spontaneously, many performers rely on external retrieval in a way that does not benefit future performance. We have also shown that combining external retrieval with previous internal retrieval attempts remediates the potential drawbacks of external retrieval and improves future performance (also see Logan & Klapp, 1991; Pyke & LeFevre, 2011). Thus, blindly relying on external retrieval without “actively” stepping in for internal retrieval might indeed be bad for us, especially in the long run. Fortunately, adding internal retrieval attempts before any external retrieval seems to be a low-cost solution.

But we caution that internal retrieval attempts might not always be beneficial. For example, when the target information is rarely needed, the benefits of internal memory might not kick in and any costs associated with internal retrieval attempts should have better been avoided. Future research could try to clearly delineate the costs of retrieval attempts. Also, exclusively storing information externally has the benefit of decreased interference with internally stored information (Storm & Stone, 2015), which might be another reason why constant internal retrieval attempts might not always be beneficial.

### Theoretical implications

Our results second earlier research (Bajic & Rickard, 2009; Bourne et al., 2006; Karpicke & Roediger, 2008; Pyke & LeFevre, 2011) by suggesting that retrieval attempts from long-term memory are not automatically initiated. That retrieval is automatically initiated was promoted by Logan’s (1989) *instance theory*, which is hard to reconcile with effects of retrieval instructions. If retrieval attempts were automatic, instructions to retrieve should be rather useless, which they clearly were not. Admittedly, the exact working mechanism of our retrieval instruction can be questioned. The idea was that people either engage in one retrieval attempt or not, and that instructions can influence this binary choice. However, retrieval instructions might also alter attentional engagement with the stimuli, leading to a stronger associative binding between problem and solution, or, similarly, multiple rather than only one instance of that binding. Two options seem tenable. Either (1) retrieval attempts are not automatic or (2) retrieval attempts are automatic and naive—i.e., without advice—users of external retrieval do a particularly poor job of attending and encoding what is relevant for subsequent retrieval in the first place.

The notion that retrieval attempts are not automatic is also compatible with an initial choice regarding the preferred strategy (Bajic & Rickard, 2009). A problem solver would only proceed to another strategy, e.g., from internal to external retrieval, if an initially preferred strategy failed to provide an answer, e.g., if an internal retrieval attempt fails. Our results are compatible with such sequential processing with an initial choice process that determines the preferred strategy and much less compatible with a parallel implementation of different strategies. Furthermore, our results are compatible with theoretical considerations that distinguish between observation and recall learning, though the study was not designed to support this categorization. The underlying idea is that observation leads to a flexible general-purpose representation whereas recall learning leads to an inflexible but highly efficient representation (Rickard & Bajic, 2006). Present results are compatible with the view that for a naive user, tech-mediated recall constitutes observation learning, which prevents establishment of inflexible stimulus-specific but highly efficient cue-target-bindings.

Lastly, the present results also relate to what has been termed “avoidance of memory retrieval” (reviewed by Touron, 2015). Such avoidance was specifically shown for older in comparison with younger adults. In the present paradigm, such avoidance would translate into a long-lasting preference of external over internal retrieval, despite a high efficiency of internal retrieval. One of the possible reasons for internal retrieval avoidance in older adults is the active choice against internal memory strategies even in situations where internal memory for the respective problem had already been mastered (Touron, 2015). Such choice might be driven by low confidence in one’s mnemonic ability, which might have been influenced by stereotypes like “memory is getting worse with age”. Interestingly, the present results indicate a pronounced avoidance of memory retrieval even for a sample of mostly young adults. Although the present sample is not suitable for testing whether avoidance is worse for older adults, it is suited for showing that retrieval avoidance can be an issue for younger adults as well. That the emphasis on older adults when investigating memory avoidance might not be warranted has also been shown in the prospective memory domain. Specifically, older adults were shown to be over- rather than underconfident in their memory ability in one study (Scarampi & Gilbert, 2021) and older adults have been shown to be well calibrated when choosing between internal memory and external alternatives, i.e., not biased toward external alternatives without a performance-related reason (Tsai et al., 2023). We conclude that avoidance of internal retrieval is not confined to older adults. Instead, retrieval avoidance might even be more problematic in younger generations who grew up with tech-mediated alternatives like the calculator and thus able to “dodge” retrieval strategies (also see LeFevre et al., 2014).

### Limitations and future research

A rather large proportion of the advice group did evidently not fully follow the strategy advice we provided. We suggested that these participants did understand the instructions but proactively decided against following them. Nevertheless, the advice group as a whole exhibited substantially improved performance in the internal block as compared to the no advice group (see Fig. 5b). Two conclusions are tenable. First, preferences for certain cognitive strategies might be so profound for some individuals that simple advice is not enough to influence their behavior. Second, a more prominent strategy advice, possibly combined with some sort of incentive, might increase the already large effect size regarding internal performance (see Fig. 5b) but possibly also regarding initial choice performance (see Fig. 5a).

The present research was designed with applicability to the human-tech interaction domain in mind. However, our main finding that relevant memory traces are not automatically established on the fly might well generalize to situations where exclusively *internal* cognitive strategies are available. Applied to the present paradigm, this would mean that advising participants to only rely on an internal counting instead of an internal retrieval strategy might or might not prohibit the establishment of internal memory (also see Pyke & LeFevre, 2011). Future research is needed to address this question.

## Conclusion

More generally, not only establishing internal memory retrieval but creating and employing any sort of cognitive strategy bears the chance to improve performance. Other researchers have called such creation of novel ways to process information a *change in the cost structure of the inferential landscape* (Kirsh, 2010) or the creation of a *novel cognitive loop* (Clark, 2011; Paul, 2021). Here, we provided evidence that human performers embedded in computerized environments can benefit from simple advice about how to best create and employ such loops. The pronounced benefit of strategy advice in the present study also suggests that there are limits to the adaptivity of the human cognitive system in technologized environments. Consequentially, the task of the responsible designer does not end with creating a cognitive environment. It ends with teaching users how to best use it.

### Supplementary Information

**Additional file 1**. Supplemental Materials.

## Data Availability

Data and stimulus materials for both experiments are available in an online repository [link will be added for publication]. The study was preregistered at https://osf.io/r8fb3 though we want to emphasize that our data quality necessitated adjustments of exclusion criteria and that our preregistered hypothesis H2 was adjusted and split into two hypotheses in the present paper because the initial analysis was substantially flawed (see Additional file 1: Fig. S3).

## Footnotes

1 More precisely, we altered the preregistered accuracy threshold and excluded participants below 75% rather than 80% accuracy in the internal block because participants performed worse than expected and we already needed to exclude 19% of participants with the more lenient criterion. In hindsight, we substantially overestimated our participants’ mental rotation abilities.

2 We anticipated the issue in the pilot stage. Hence, we implemented several measures to ensure that participants knew about the strategy advice. With the present measures in place (see Methods), we are confident that some participants deliberately decided to not follow our strategy advice rather than missed the relevant instructions. This is also reflected in informal postexperimental interviews in which several participants stated that they simply *wanted to count* despite the instructions, which might be some variant of mental challenge seeking.

3 When choosing a more conservative criterion and only including participants in the advice condition who used external retrieval in more than half of the first 12 trials, the effect further strengthens; *M*_advice_ = 1592 ms_,_ *M*_no advice_ = 1962 ms, *t*(72) = 2.95, *p* < .002, *d* = .73, CI95_*d*_ = [.27 1.15].

4 Results are similar when excluding the sixteen participants who did not obey advice; *M*_advice_ = 1201 ms_,_ *M*_no advice_ = 1790 ms, *t*(80) = 3.47, *p* = .0004, *d* = .79, CI95_*d*_ = [.34 1.19]. Because of that and because there is evidence that these participants at least partially started obeying instructions over time (Additional file 1: Fig. S2b), we decided to use the full sample for this analysis.

5 The statistical pattern stayed identical without filtering any participants; *p* = .010 versus *p* = .192. Note that 34 participants did not exhibit a single trial with an RT* > 2000 ms during the internal choice block, naturally resulting in a lower sample size for even the unfiltered analysis.
