# Supplementary material for: Overreliance on inefficient computer-mediated information retrieval is countermanded by strategy advice that promotes memory-mediated retrieval
Source: Cogn Res Princ Implic. 2023 Dec 20;8:72. doi: 10.1186/s41235-023-00526-6 (PMC10733273; doi:10.1186/s41235-023-00526-6)
Supplement: Supplementary file 1 — Additional file 1. Supplemental Materials. [file 41235_2023_526_MOESM1_ESM.docx]

# Supplemental Materials

**Figure S1**

*External Retrieval During First 12 Trials of Choice Block*


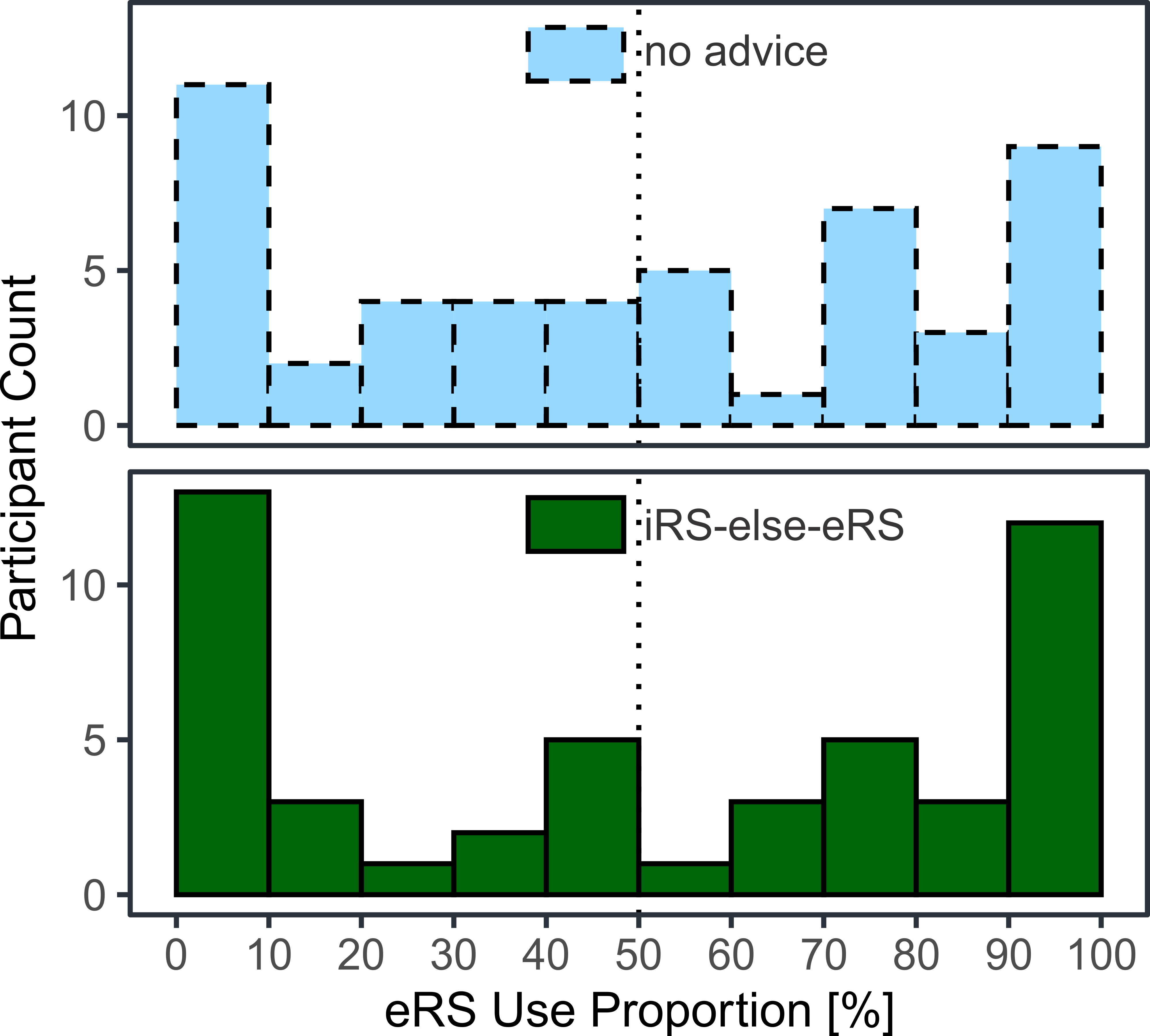


*Note*. eRS = external Retrieval Strategy.

**Figure S2**

*External Retrieval over Time depending on Initial External Retrieval*


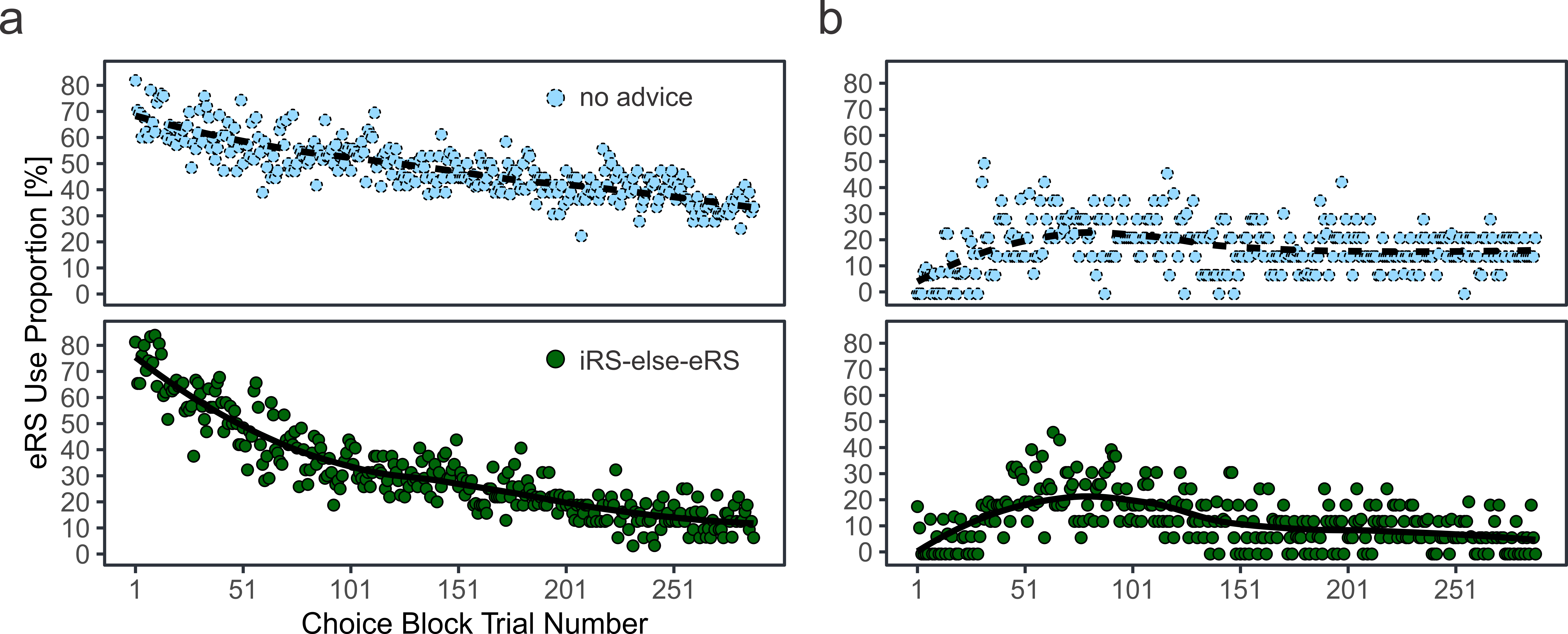


*Note*. Participants who used the eRS in at least four of the first twelve trials exhibited a high level of eRS use that declined over time (a). Conversely, participants who used the eRS at most three times during the first twelve trials exhibited a small increase in eRS use (b). Crucially, only participants in the iRS-else-eRS advice group returned to low eRS use proportions thereafter, suggesting that these participants at least partially followed the iRS-else-eRS advice.

eRS = external Retrieval Strategy, iRS = internal Retrieval Strategy.

**Figure S3**

*Effects of Addend on Internal Block Accuracy and RT*


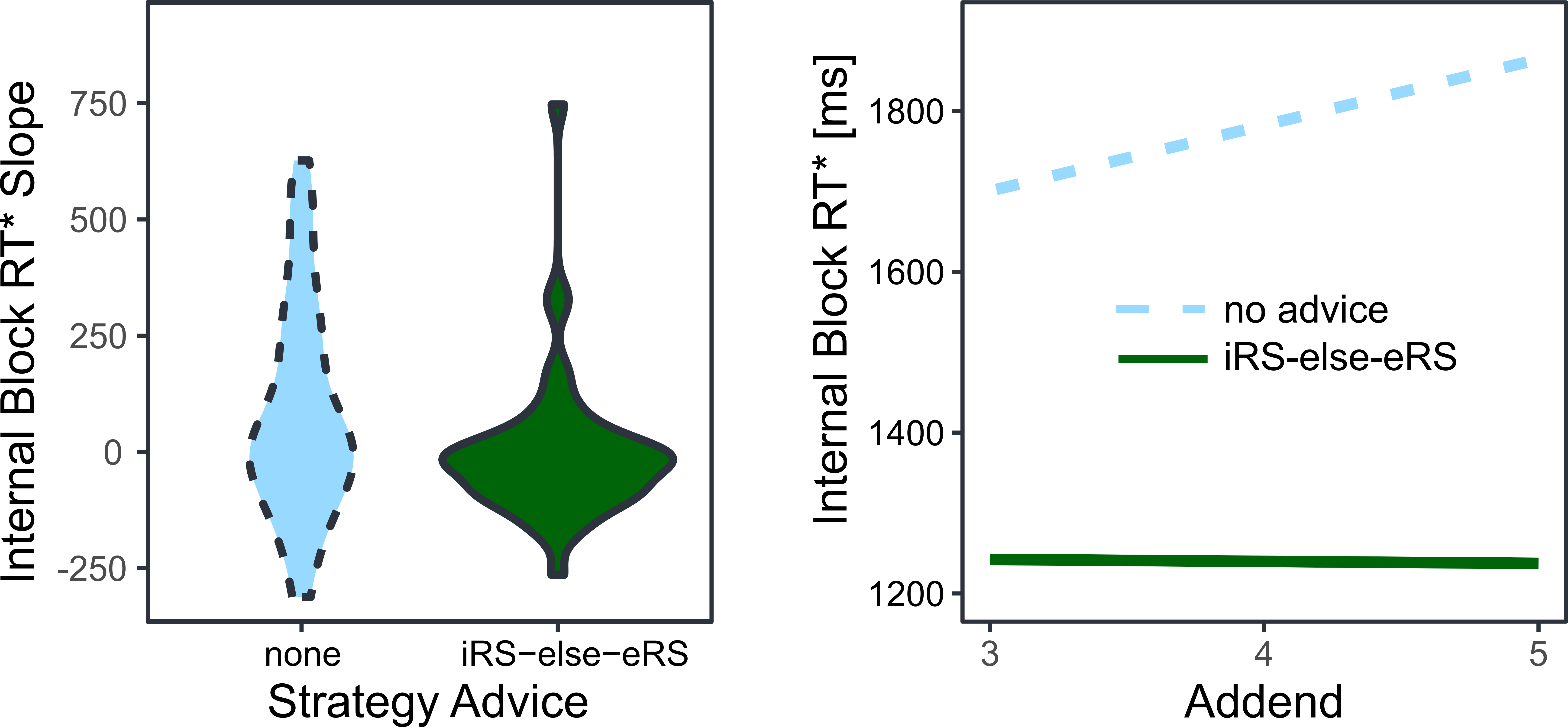


*Note*. We calculated a linear model for each participant, predicting RT* in the internal block from addend. A one-sided independent *t*-test revealed that the slope was higher for the no advice in comparison to the iRS-else-eRS group; *M*_advice_ = 2 ms _,_ *M*_no advice_ = 82 ms, *t*(96) = 2.11, *p* = .019, *d* = .43, CI95*_d_* = [-.02 .80]. However, since it remains unclear whether the higher slope in the no advice group was caused by worse internal memory or by increased selection of the counting strategy, we excluded this analysis from the main manuscript.

**Post-Experimental Questions [in German]**

1. Demographic questions
   1. Wie alt sind Sie (in Jahren)?
   2. Bitte geben Sie Ihr Geschlecht an.
      - Weiblich, Männlich, Divers, Ich möchte keine Angabe machen
   3. Sprechen Sie flüssig Deutsch?
      - Ja, Nein
   4. Können Sie normal sehen (weil Sie keine Sehbeschwerden haben bzw. die Beschwerden durch z.B. Brillle ausgeglichen sind)?
      - Ja, Nein
   5. Führen sie alltägliche Tätigkeiten bevorzugt mit einer bestimmten Hand aus?
      - Ja: rechte Hand, Ja: linke Hand, Nein
   6. Wie geübt sind Sie im Umgang mit Computermäusen?
      - Sehr ungeübt,,ausgeglichen,,Sehr geübt (0-4)
2. Metacognitive questions
   1. Für welche Strategie haben Sie sich im letzten Teil des Experiments, als die schwarze Box deaktiviert war, entschieden? [metafq2_slid]

*Hochzählen beinhaltet das bewusste Hochzählen des Alphabets. Gedächtnis beinhaltet sowohl das sichere Abrufen der Lösung aus dem Gedächtnis als auch ein gutes Bauchgefühl.*

- ausschließlich Gedächtnis, , ausgeglichen, , ausschließlich Hochzählen (1-5)
  1. Welche Strategie haben Sie in den Teilen des Experiments, in denen Sie die freie Wahl zwischen Gedächtnis, Hochzählen, und Maus hatten, meistens benutzt?
     - Gedächtnis, Hochzählen, Maus
  2. Haben Sie darauf geachtet, die Anweisungen zur Wahl der Strategien (Gedächtnis, Hochzählen, Maus) zu befolgen?
     - nein, , mal so mal so, , ja (0-4)
  3. Mit welcher Strategie haben Sie am schnellsten geantwortet?
     - Gedächtnis, Hochzählen, Maus
  4. Mit welcher Strategie haben Sie am korrektesten geantwortet?
     - Gedächtnis, Hochzählen, Maus
  5. Wie oft haben Sie mehrere Strategien gleichzeitig (z.B. Hochzählen und Maus) angewendet?
     - nie, , mal so mal so, , immer (0-4)
  6. Wie wichtig war es Ihnen, während der Studie gute Leistungen zu erbringen?
     - nicht sehr wichtig, , ausgewogen, , sehr wichtig (0-4)
  7. Sie haben gerade ausgewählt, für welche Strategie (Gedächtnis, Hochzählen, oder Maus) sie sich am Häufigsten entschieden haben. Warum haben Sie sich so entschieden?

*Bitte tippen Sie Ihre Antwort auf der Tastatur in ein bis drei Sätzen ein.*

- 1. German Need For Cognition Scale (Bless et al., 1994; Cacioppo & Petty, 1982) [16 items]
